# Lipids and glucose fluctuations are associated with clinically diagnosed depression in older adults with type 2 diabetes

**DOI:** 10.3389/fendo.2026.1733766

**Published:** 2026-06-02

**Authors:** Hao Geng, Yan Sun, Xiaoming Kong, Siwen Lv, Letian Yang, Xiaomin Hu, Hong Hong, Nannan Zhu, Li Zhang, Yangliu Pei, Yu Guo, Pengyu Xie, Zifan Zhu

**Affiliations:** 1 Affiliated Psychological Hospital of Anhui Medical University, Hefei, China; 2 Department of Geriatric Psychology, Hefei Fourth People’s Hospital, Hefei, China; 3 Anhui Mental Health Center, Hefei, China; 4 Anhui Clinical Research Center for Mental Disorders, Hefei, China; 5 The Fifth Clinical College of Anhui Medical University, Hefei, China; 6 The First Affiliated Hospital of Anhui Medical University, Hefei, China

**Keywords:** dyslipidemia, elderly patients, glycemic variability, major depressive disorder, T2DM

## Abstract

**Introduction:**

Major depressive disorder (MDD) frequently occurs in elderly patients with type 2 diabetes (T2D), amplifying the overall disease burden. While lipid and glucose fluctuations have been suggested as contributors to MDD, their precise roles in clinically diagnosed secondary depression require further elucidation. This study aimed to identify key indicators related to lipids and glycemic levels associated with depression in older adults with type 2 diabetes.

**Methods:**

We retrospectively analyzed data from 145 hospitalized T2D patients aged 60 years or older, including 86 with MDD and 59 without MDD. We gathered information on their lipid profiles (e.g., total cholesterol [TC], apolipoprotein B [ApoB]) and glucose fluctuations (e.g., standard deviation of blood glucose [SDBG], low blood glucose index [LBGI], time in range [TIR]) using continuous glucose monitoring (CGM). Univariate and multivariate logistic regression analyses were conducted to identify factors independently associated with clinically diagnosed depression.

**Results:**

The prevalence of comorbid MDD among hospitalized older adults with T2D was 59.31% (86/145). Correlation analysis demonstrated significant associations between glycemic variability, lipid parameters, and psychological measures. In multivariable logistic regression, ApoB (OR = 15.311, 95% CI 2.688, 1on,le p = 0.002), SDBG (OR = 1.409, 95% CI 1.121= 1on,l p = 0.003), and LBGI (OR = 1.327, 95% CI 1.101= 1on,l p = 0.003) remained independently associated with comorbid MDD.

**Conclusion:**

In this study, the prevalence of comorbid MDD among hospitalized older T2D patients was 59.31% (86/145). Abnormal lipid levels and glucose fluctuations are strongly associated with MDD in older T2D patients. ApoB, SDBG, and LBGI may serve as potential clinical markers associated with comorbid MDD. Further larger prospective studies are necessary to confirm these findings and inform clinical management.

## 1 Introduction

Major depressive disorders (MDD) are highly prevalent among individuals with diabetes, affecting an average of one in four patients—a rate two to three times higher than in the general population. The prevalence of MDD is even greater in specific age groups, such as older adults with diabetes (1). MDD is a serious complication that leads to poorer medication adherence, reduced quality of life (2), worse prognosis (3), and higher mortality (4) in diabetic patients. Identifying type 2 diabetes (T2D) patients who are more likely to present with MDD is clinically relevant for the long-term management of the disease, especially in older patients with multiple comorbidities.

The occurrence of depression in patients with T2D has been reported to be associated with a variety of factors. In China, the prevalence of depression among adults with T2D was reported to be 25.9%, with associated variables including gender, age, duration of diabetes, and comorbidities (5). However, the prevalence of depression and its associated risk factors in patients with T2D vary across countries and age groups (1, 6, 7). Depression in T2D is linked to poor glycemic control and self-management, particularly in older adults with multimorbidity. Identifying risk factors in this population is essential for early detection and intervention (8).

Both dyslipidemia and abnormal glucose fluctuations have been associated with poor prognosis in patients with diabetes mellitus and depression. However, there is a research gap regarding the combined effect of these factors on secondary depression in elderly patients with T2D. A study of elderly T2D patients indicated that high HbA1c levels, cardiovascular disease, and retinopathy were strongly associated with mild cognitive impairment (MCI), while factors such as female gender, single marital status, smoking history, physical inactivity, higher body mass index (BMI), and elevated total cholesterol were major risk factors for depression (9). A cohort study involving 151,814 Korean subjects with data from at least three follow-up visits suggested that persistent poor glycemic control, such as high glycemic variability (GV), is an important risk factor for depression and anxiety disorders (10). Collectively, these findings indicate that both lipid levels and glycemic fluctuations are important metabolic characteristics observed in patients with comorbid depression in elderly patients with T2D. However, previous studies have often included limited glycemic metabolic indices and have not formally evaluated statistical interaction effects between glycemic and lipid parameters.

Over the past decade, evidence has demonstrated a bidirectional association between T2D and depression, with a higher prevalence of depressive symptoms in older adults with diabetes. However, most studies rely on screening tools rather than clinically confirmed MDD. Dyslipidemia and glycemic variability have both been linked to adverse psychological outcomes, yet these factors are typically studied in isolation. Given the complex metabolic profile of elderly patients with T2D, integrated analyses of lipid parameters and glucose variability in relation to clinically diagnosed MDD remain limited.

Despite growing evidence linking metabolic abnormalities and depressive symptoms in diabetes, several important gaps remain. First, most prior studies have relied on self-reported depressive symptom scales rather than clinically confirmed MDD diagnoses based on standardized psychiatric criteria. Second, many investigations have examined either lipid parameters or glycemic indices in isolation, without comprehensively evaluating a broad range of lipid markers together with continuous glucose monitoring–derived variability metrics within the same elderly diabetic cohort. Third, data focusing specifically on hospitalized older adults with T2D—who often present with multimorbidity and complex metabolic profiles—remain limited. Therefore, the present retrospective study aimed to jointly examine lipid metabolism markers and glycemic variability indices in relation to clinically diagnosed MDD among hospitalized older adults with T2D. Rather than assessing statistical interaction effects, our objective was to identify metabolic variables independently associated with comorbid MDD within this specific clinical population.

## 2 Participants and methods

### 2.1 Participants

We retrospectively included 145 hospitalized patients aged ≥ 60 years with confirmed T2D who were admitted between January 2020 and April 2023. Among them, 86 patients met DSM-5 diagnostic criteria for MDD and had a HAMD-24 score ≥ 20 (T2D + MDD group), while 59 patients had T2D without a diagnosis of MDD (T2D-only group).

All participants met the diagnostic criteria for T2D according to the 2020 American Diabetes Association (ADA) guidelines (11) (fasting plasma glucose ≥ 7.0 mmol/L or 2-hour plasma glucose ≥ 11.1 mmol/L).

#### 2.1.1 Inclusion criteria (applied to all participants)

① Age ≥ 60 years.
② Diagnosis of T2D according to 2020 ADA criteria.
③ Ability to cooperate with laboratory testing and questionnaire assessments.

Additional inclusion criterion for the MDD group:

④ Diagnosis of MDD according to DSM-5.
⑤ HAMD-24 score ≥ 20.

#### 2.1.2 Exclusion criteria (applied to all participants)

① Age< 60 years.
② Diagnosis of type 1 diabetes mellitus.
③ Severe acute diabetic complications (e.g., ketoacidosis, hyperosmolar hyperglycemic state).
④ Severe organic diseases.
⑤ Use of medications affecting lipid metabolism within the past three months.
⑥ History of bipolar disorder or other major psychiatric disorders (except MDD in the MDD group).

The only difference between groups was the presence or absence of clinically diagnosed MDD.

### 2.2 Clinical data collection

All participants underwent comprehensive data collection, including laboratory tests, continuous glucose monitoring (CGM), and assessments using HAMD-24 and HAMA scales. These evaluations were conducted by three licensed and experienced clinical psychiatrists. The study adhered to ethical standards and was approved by the Ethics Committee of Anhui Medical University Affiliated Psychological Hospital. Written informed consent was obtained from all participants or their legal guardians.

### 2.3 Laboratory assessments

After fasting for 8–12 hours, venous blood samples were collected from participants in the morning. A fully automated biochemical analyzer (Roche c8000) was used to measure lipid metabolism indicators, including total cholesterol (TC), high-density lipoprotein (HDL), apolipoprotein A (ApoA), apolipoprotein B (ApoB), and triglycerides (TG). The reference ranges were as follows: Total cholesterol (2.86 – 5.18 mmol/L), ApoA1 (1.00 – 1.60 g/L), ApoB (0.60 – 1.19 g/L), Triglycerides (0.56 – 1.70 mmol/L).

### 2.4 Continuous glucose monitoring

All participants underwent continuous glucose monitoring (CGM) after enrollment. The monitoring device was worn for at least 72 hours, providing interstitial glucose measurements at 5-minute intervals (approximately 864 data points per participant). The CGM system used was the Abbott FreeStyle Libre H.

During the monitoring period, meal timing was standardized, and glucose-lowering treatment regimens were maintained without modification. After completion of monitoring, glucose data were extracted using the CGM report management system.

The following glycemic variability indices were calculated:

① Coefficient of Variation (CV): CV=SD/MBG×100%
② Estimated HbA1c (eHbA1c): eHbA1c(%)=(MBG+46.7)/28.7
③ Area Under the Curve (AUC): $\text{AUC}=\sum_{\text{i}=1}^{\text{n}-1}\frac{\left({\text{G}}_{\text{i}}+{\text{G}}_{\text{i}+1}\right)}{2}\times ({\text{t}}_{\text{i}+1}-{\text{t}}_{\text{i}})$
④ High Blood Glucose Index (HBGI) and.
⑤ Low Blood Glucose Index (LBGI):
$$\text{f}(\text{G})=1.509\times [{\left(\ln\text{G}\right)}^{1}.084-5.381]$$
$$\text{r}(\text{G})=10\times {\left[\text{f}(\text{G})\right]}^{2}$$
$$\text{HBGI}=\frac{1}{\text{n}}\sum \text{r}({\text{G}}_{\text{i}}),\text{ for f}({\text{G}}_{\text{i}})>0$$
$$\text{LBGI}=\frac{1}{\text{n}}\sum \text{r}({\text{G}}_{\text{i}}),\text{ for f}({\text{G}}_{\text{i}})<0$$
⑥ Mean Blood Glucose (MBG): MBG=(1/n)ΣGi

### 2.5 Assessment of depressive and anxiety symptoms

The 24-item Hamilton Depression Rating Scale (HAMD- 24) and the Hamilton Anxiety Scale (HAMA) were used to assess the severity of depressive and anxiety symptoms, respectively. HAMD- 24 includes items scored on either a 5- point Likert scale (0 – 4) or a 3- point scale (0 – 2), while all items in HAMA are scored on a 5-point scale (0 – 4). HAMD- 24 Scoring Interpretation: 0–8 points: No depressive symptoms; 20–35 points: Mild to moderate depression; > 35 points: Severe depression; HAMA Scoring Interpretation: 0–7 points: No anxiety symptoms; 7–14 points: Possible anxiety; 14–21 points: Definite anxiety; 21–29 points: Significant anxiety; > 29 points: Possible severe anxiety.

### 2.6 Statistical analysis

Statistical analyses were performed using SPSS version 26.0 (IBM Corp., Armonk, NY, USA) and R studio (version 4.4.2, https://www.r-project.org/). Continuous data were tested for normality using the Shapiro-Wilk test. Normally distributed data were expressed as mean ± standard deviation (SD) and compared using independent sample Students’ tests (t- test). Categorical data were expressed as frequencies and percentages, and group comparisons were made using the chi-squared (χ²) test. Correlation analyses were conducted using Pearson’s correlation coefficient for normally distributed variables. Multiple linear regression analysis was performed to identify independent predictors of outcomes. Variables meeting the predefined univariate threshold (P< 0.10) were entered into the multivariable model. Variables showing P< 0.10 in univariate logistic regression were considered for inclusion in the multivariable logistic regression model. In addition, age, sex, BMI, and T2D duration were included as covariates based on clinical relevance, regardless of univariate significance. A two-tailed p-value of less than 0.05 was considered statistically significant. To facilitate comparison of effect sizes across variables, standardized logistic regression models were additionally performed by converting continuous predictors to z-scores.

## 3 Results

### 3.1 Study population

A total of 145 patients with T2D were included in the final data analysis. There were no significant differences between groups in age, sex distribution, height, weight, or BMI (all P > 0.05). Quantitative data were presented as mean ± S.D., while qualitative data were expressed as n (%). Among the participants, 59 patients were diagnosed solely with T2D, and 86 patients were diagnosed with both T2D and MDD. Compared to patients with T2D alone, those with T2D and MDD exhibited significantly higher HAMD scores (*T* = 35.442, *P* < 0.001), and HAMA scores (*T* = 7.745, *P* < 0.001). Detailed results are presented in **Table 1**.

**Table 1:** Characteristics of study population.

Variables	T2D (n=59)	T2D with MDD (n=86)	T value/χ^2^	p
Age (years)	70.24 ± 7.54	72.42 ± 5.75	1.975	0.063
Sex, male n (%)	31 (52.5%)	43 (50.0%)	0.09	0.76
Height (cm)	159.36 ± 9.25	158.36 ± 9.76	0.619	0.537
Weight (kg)	61.04 ± 10.65	60.02 ± 10.81	0.561	0.576
BMI (kg/m^2^)	24.22 ± 4.81	23.80 ± 2.99	0.647	0.553
T2D duration (months)	141.41 ± 108.67	155.72 ± 126.07	0.710	0.479
MDD duration (months)	–	60.85 ± 53.02	8.806	<0.001
HAMD score	3.78 ± 1.98	31.98 ± 6.98	35.442	<0.001
HAMA score	3.05 ± 1.92	24.03 ± 6.62	27.745	<0.001

Continuous variables are presented as mean ± SD; categorical variables as n (%). Differences between groups were assessed using independent t-tests or chi-square tests, as appropriate.

BMI, Body mass index; MDD, Major depressive disorders; T2D, type 2 diabetes; HAMD, Hamilton Depression Rating Scale; HAMA, Hamilton Anxiety Scale.

### 3.2 Significant differences in lipid metabolism and glycemic variability indicators between T2D patients with and without MDD

We analyzed the lipid metabolism and glycemic variability indicators between T2D patients with and without MDD and observed significant differences between the two groups.

Compared to patients with T2D alone, those with both T2D and MDD had significantly higher levels of TC (*T* = 3.985, *P* < 0.001), HDL (*T* = 2.927, *P* = 0.003), ApoA (*T* = 4.974, *P* < 0.001), and ApoB (*T* = 4.073, *P* < 0.001). However, no significant difference was found in TG levels (*T* = 0.277, *P* = 0.782).

**Figure 1:**
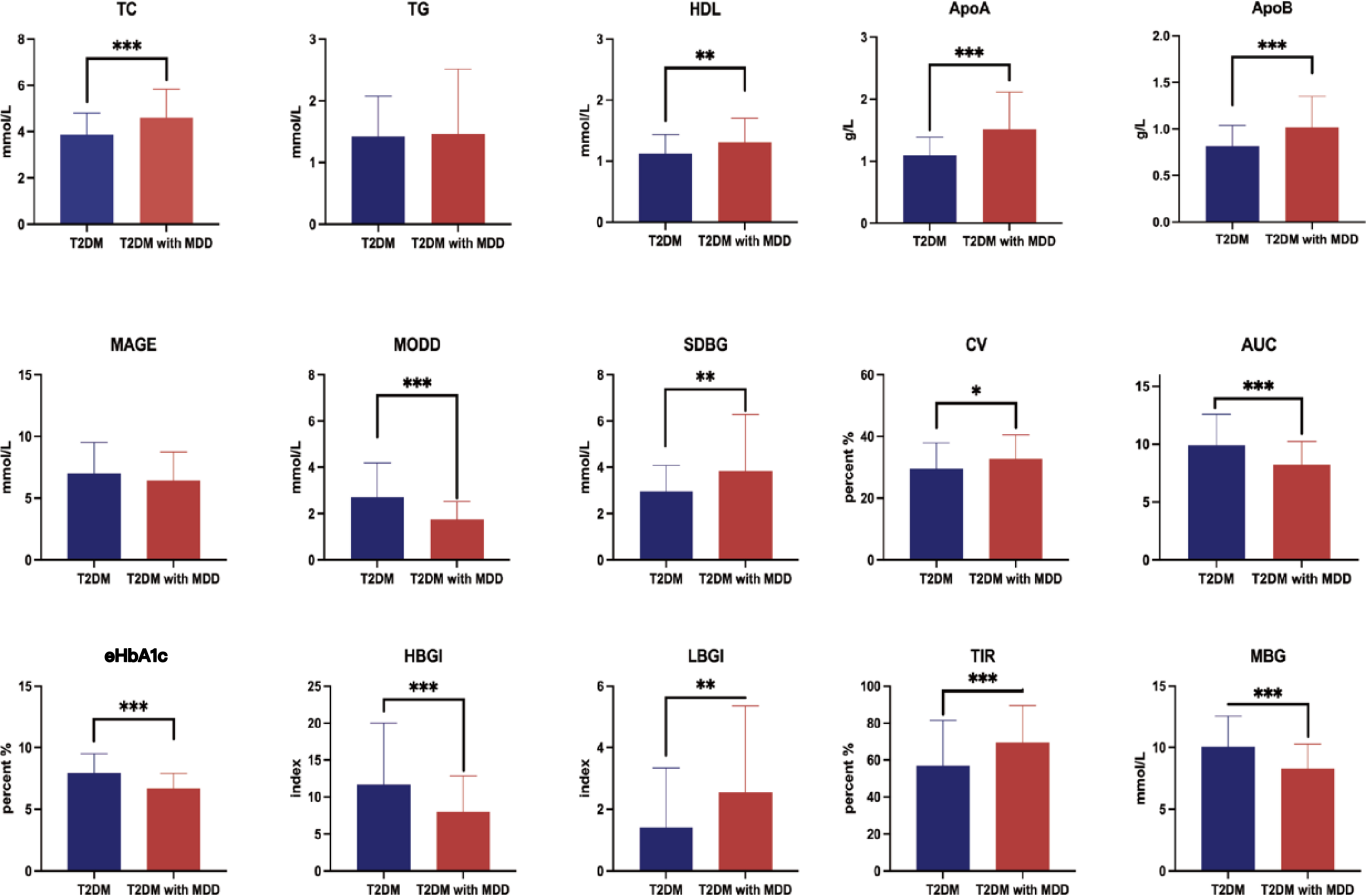
Differences in lipid metabolism and glycemic variability indicators between T2DM patients with and without MDD. This figure illustrates the significant differences in lipid metabolism (TC, HDL, ApoA, ApoB) and glycemic variability indicators (SDBG, CV, LBGI, TIR, MODD, AUC, eHbA1c, HBGI, MBG) between T2DM patients with and without MDD. Among T2DM patients with MDD, levels of TC, HDL, ApoA, ApoB, SDBG, CV, LBGI, and TIR were significantly higher compared to those without MDD, whereas MODD, AUC, eHbA1c, HBGI, and MBG were significantly lower. No significant differences were observed for TG and MAGE. Statistical significance is indicated with relevant markers. Data are presented as mean ± standard deviation (SD). Major depressive disorder (MDD) , type 2 diabetes (T2D), total cholesterol (TC), apolipoprotein B (ApoB) , glucose fluctuations, standard deviation of blood glucose (SDBG), low blood glucose index (LBGI), time in range (TIR). *p<0.05, **p<0.01,***p<0.001

Similarly, glycemic variability indicators showed notable differences between the groups. Patients with T2D and MDD had significantly higher SDBG (*T* = 2.583, *P* = 0.011), CV (*T* = 2.190, *P* = 0.03), LBGI (*T* = 2.671, *P* = 0.008), and TIR (*T* = 3.364, *P* < 0.001). Conversely, these patients exhibited significantly lower levels of MODD (*T* = 5.029, *P* < 0.001), AUC (*T* = 4.377, *P* < 0.001), eHbA1c (*T* = 5.308, *P* < 0.001), HBGI (*T* = 3.369, *P* < 0.001), and MBG (*T* = 4.620, *P* < 0.001). However, no significant difference was observed in MAGE (*T* = 0.277, *P* = 0.782). The detailed results are presented in **Figure 1**.

### 3.3 Correlations among glycemic variability, lipid metabolism, and psychological indicators

**Figure 2:**
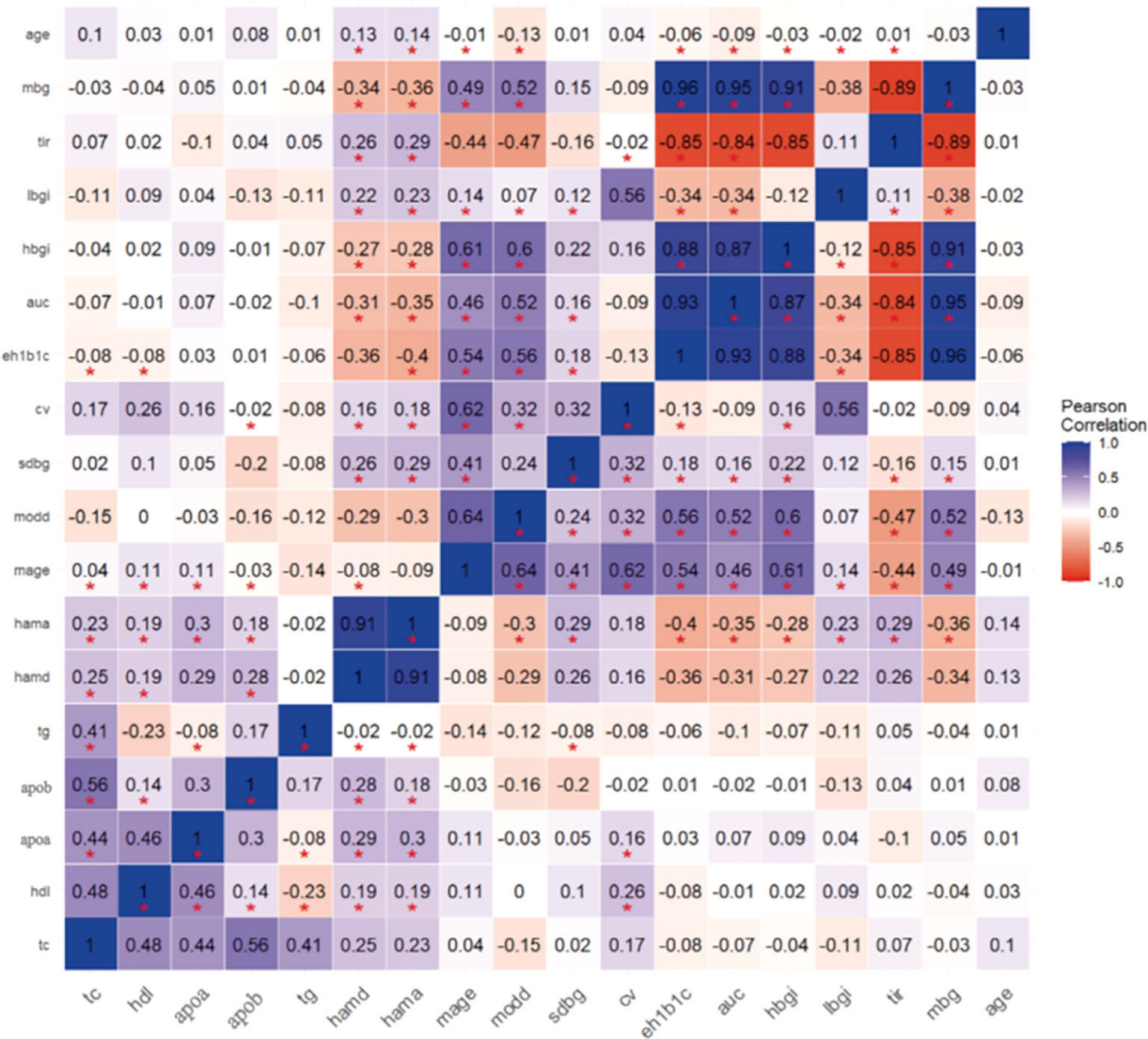
Correlation heatmap of glycemic variability, lipid metabolism, and psychological indicators in T2DM patients. Heatmap of Pearson correlation coefficients among glycemic variability indices (MBG, TIR, CV), lipid metabolism markers (TG, HDL, ApoA, ApoB), and psychological measures (HAMA, HAMD) in patients with T2D. Strong positive correlations were observed between eHbA1c and MBG (R = 0.96, P< 0.01) as well as AUC (R = 0.93, P< 0.01), and a strong negative correlation was found between eHbA1c and TIR (R = –0.85, P< 0.01). HDL showed modest positive correlations with HAMA and HAMD scores (R = 0.19 for both, P< 0.05), whereas ApoB was positively correlated with HAMD (R = 0.28, P< 0.05). Statistical significance is indicated by * (P< 0.05). MDD, Major depressive disorder; T2D, type 2 diabetes; TC, total cholesterol; ApoB, apolipoprotein B; SDBG, glucose fluctuations, standard deviation of blood glucose; LBGI, low blood glucose index; TIR, time in range.

The Pearson correlation heatmap (**Figure 2**) illustrates the associations among glycemic variability indices, lipid metabolism markers, and psychological measures in patients with T2D.

Strong correlations were observed among key glycemic parameters. eHbA1c was strongly positively correlated with MBG (R = 0.96, P< 0.01) and AUC (R = 0.93, P< 0.01), and strongly negatively correlated with TIR (R = –0.85, P< 0.01).

Regarding lipid and psychological variables, HDL showed modest positive correlations with both HAMA and HAMD scores (R = 0.19 for both, P< 0.05). In contrast, ApoB was positively correlated with HAMD scores (R = 0.28, P< 0.05).

These results demonstrate statistically significant associations between selected glycemic, lipid, and psychological variables in this cohort of patients with T2D.

### 3.4 Factors independently associated with comorbid MDD in patients with T2D

Binary logistic regression was performed with comorbid MDD status as the dependent variable. In univariate analyses, TC [OR = 1.882, 95% CI (1.338, 2.647), P< 0.001], ApoB [OR = 11.932, 95% CI (3.199, 44.503), P< 0.001], SDBG [OR = 1.284, 95% CI (1.049, 1.570), P = 0.015], and LBGI [OR = 1.239, 95% CI (1.047, 1.467), P = 0.013] were significantly associated with MDD.

A multivariable logistic regression model was then constructed, additionally adjusting for age, sex, BMI, and T2D duration to account for potential confounding. After adjustment, ApoB [OR = 15.311, 95% CI (2.688, 87.205), P = 0.002], SDBG [OR = 1.409, 95% CI (1.121, 1.771), P = 0.003], and LBGI [OR = 1.327, 95% CI (1.101, 1.601), P = 0.003] remained independently associated with MDD, whereas the association of TC was attenuated and no longer statistically significant [OR = 1.538, 95% CI (0.971, 2.436), P = 0.066].

**Figure 3:**
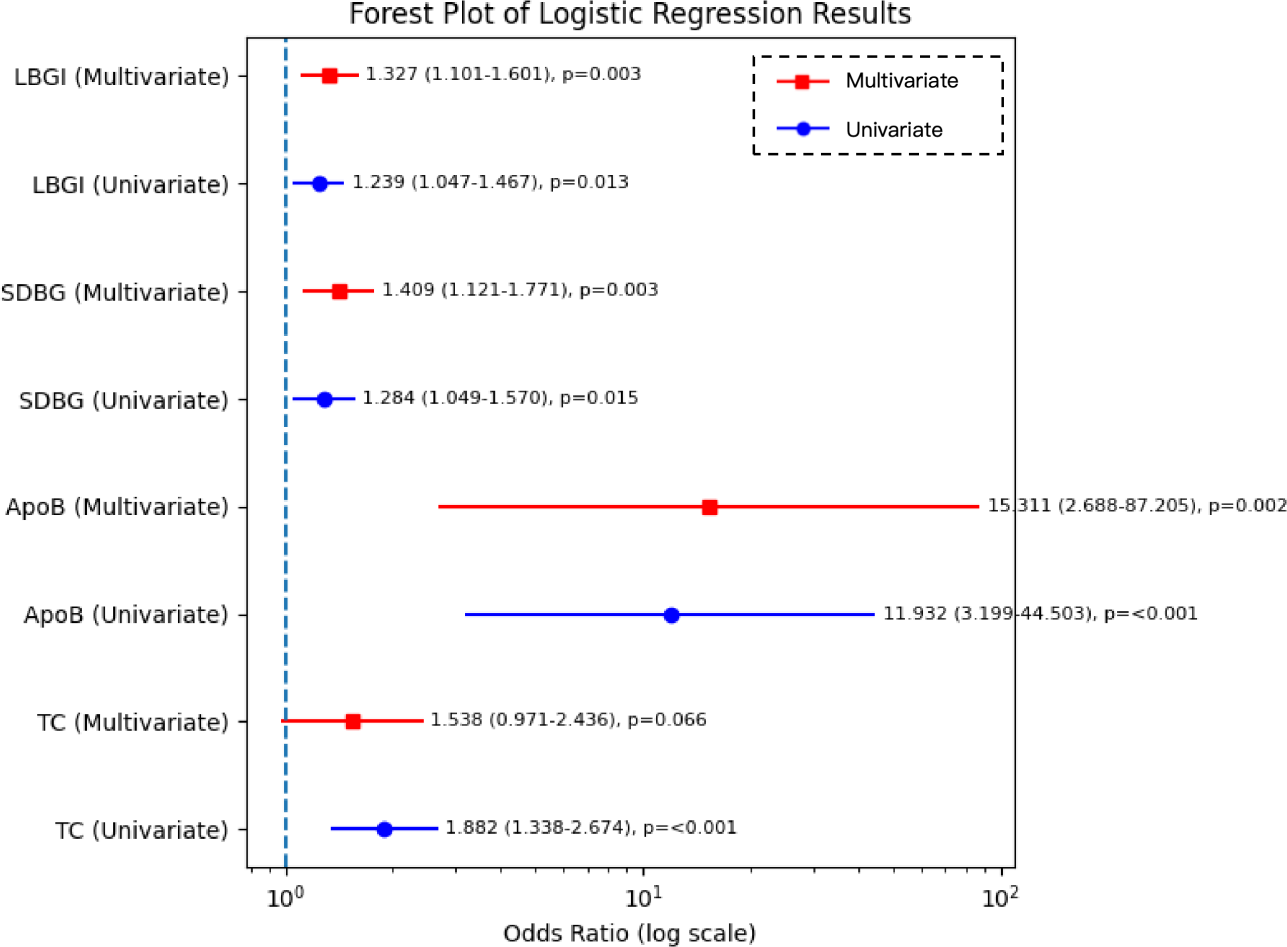
Forest plot of univariate and multivariate logistic regression analyses of factors associated with comorbid MDD in patients with T2D. Odds ratios (ORs) and 95% confidence intervals (CIs) are presented for TC, ApoB, SDBG, and LBGI. The multivariable model was adjusted for age, sex, BMI, and T2D duration. Error bars represent 95% CIs, and the vertical dashed line indicates OR = 1. Corresponding OR values, confidence intervals, and p-values are annotated alongside each estimate.

Standardized regression analyses showed that a one–standard deviation increase in ApoB, SDBG, and LBGI was associated with ORs of 2.31, 2.02, and 2.06, respectively. The regression results are presented in **Figure 3**, and the fully adjusted model is summarized in **Table 2**.

**Table 2:** Unadjusted and adjusted logistic regression analyses of factors associated with comorbid MDD in patients with T2D.

Predictor	Unadjusted OR (95% CI)	P	Adjusted OR (95% CI)	P_adj
TC	1.882 (1.338, 2.647)	<0.001	1.538 (0.971, 2.436)	0.066
ApoB	11.932 (3.199, 44.503)	<0.001	15.311 (2.688, 87.205)	0.002
SDBG	1.284 (1.049, 1.570)	0.015	1.409 (1.121, 1.771)	0.003
LBGI	1.239 (1.047, 1.467)	0.013	1.327 (1.101, 1.601)	0.003
Age	—	—	1.052 (0.990, 1.119)	0.105
Sex (male)	—	—	1.198 (0.484, 2.963)	0.696
BMI	—	—	0.961 (0.861, 1.072)	0.473
T2D duration	—	—	1.001 (0.997, 1.004)	0.728

Adjusted for age, sex, BMI, and T2D duration. Unadjusted ORs were derived from separate univariate logistic regression models.

MDD, Major depressive disorder; T2D, type 2 diabetes; TC, total cholesterol; ApoB, apolipoprotein B; SDBG, glucose fluctuations, standard deviation of blood glucose; LBGI, low blood glucose index; TIR, time in range.

## 4 Discussion

Our findings highlight a strong relationship between lipids, glucose fluctuations, and depression in older T2D. The key factors were ApoB, SDBG, and LBGI. These measures stood out in both univariate and multivariate analyses. Prior work focused on mild depressive symptoms or self-reported measures. We addressed that gap by examining patients who had clinically diagnosed MDD.

We observed that TC, HDL, ApoA, and ApoB levels were significantly higher in T2D patients with MDD compared to those without MDD, consistent with prior research linking lipid dysregulation to depression (12). This may be due to the fact that patients with depressive disorders are in a prolonged state of low mood, and their bodies are in a state of stress, and this stress-induced change in hormone levels increases serum cholesterol and other lipid metabolism indicators (13). Dyslipidemia may contribute to the development of depressive symptoms through pathways involving systemic inflammation, oxidative stress, and alterations in neurotransmitter synthesis (14). ApoB is an established marker of atherogenic lipoprotein particle number and cardiovascular risk, and was independently associated with MDD (15). In our study, further supporting its role as a potential biomarker for depression in diabetic patients. This finding aligns with studies suggesting that ApoB reflects atherogenic lipid burden (16), which has been implicated in both metabolic and psychological disorders (17).

Regarding lipid-related findings, ApoB was independently associated with comorbid MDD, which is consistent with ApoB being a direct marker of atherogenic lipoprotein particle number and overall lipid burden. Interestingly, ApoA also showed a positive correlation with MDD in our cohort. This observation does not necessarily imply a protective role of ApoA/HDL in this setting, as HDL-related markers may capture heterogeneous biological states in older adults with metabolic and inflammatory burden. For example, under chronic inflammation and metabolic stress, HDL particles may undergo compositional and functional alterations (so-called “dysfunctional HDL”), and ApoA-related measures may not fully reflect HDL functionality. In addition, medication use, nutritional status, and acute illness in hospitalized patients may influence apolipoprotein concentrations. Collectively, these considerations suggest that, in older hospitalized T2D patients, apolipoprotein profiles may reflect complex metabolic and inflammatory states associated with depression, and future studies incorporating more detailed lipoprotein phenotyping and functional HDL assays are warranted.

In this study, we used continuous glucose monitoring technology to assess the glucose fluctuations instead of simple evaluation method. Our results demonstrate that indicators of glycemic variability, including the SDBG and the LBGI, were significantly associated with comorbid MDD. These findings are consistent with previous studies emphasizing the impact of glycemic fluctuations on mental health outcomes (10). Poor glycemic control has been reported to be associated with psychological distress through mechanisms such as hypoglycemia-induced neurotoxicity, inflammatory responses (18), and hypothalamic-pituitary-adrenal (HPA) axis dysregulation (19). The observed negative correlation between TIR and MDD further supports the hypothesis that stable glycemic control may be associated with lower depressive symptom burden in diabetic patients. Furthermore, in elderly patients with T2D, MDD has been associated with increased cardiovascular burden (20, 21), suggesting the need to adequately plan for concomitant cardiovascular events in such patients.

In addition to indices reflecting glucose fluctuation, our results also showed that parameters reflecting greater exposure to lower glucose levels (higher LBGI and lower mean glucose/eHbA1c) were associated with comorbid MDD. This pattern is plausible in hospitalized older adults with T2D and may reflect several non-mutually exclusive clinical scenarios. First, depression in late life is often accompanied by reduced appetite, irregular dietary intake, and weight loss, which may increase susceptibility to lower glucose excursions under ongoing glucose-lowering therapy. Second, older patients with depressive symptoms may have impaired self-care behaviors and poorer adherence to structured meal plans, potentially increasing glycemic instability in both directions. Third, this pattern may also reflect “over-treatment” or overly tight glycemic targets in vulnerable older adults, in whom hypoglycemic exposure may occur despite apparently lower average glucose indices. Importantly, hypoglycemia and fear of hypoglycemia have been associated with psychological distress and reduced quality of life, which may further complicate the metabolic–mental health relationship in this population. Therefore, our findings underscore the need for individualized glycemic management in older T2D patients, particularly when depressive symptoms are present.

To our knowledge, this is the first study to comprehensively examine the combined impact of lipid metabolism and glycemic variability on the risk of MDD in elderly T2D patients. Our findings indicate that lipid metabolism and glycemic variability are both independently associated with MDD, although formal statistical interaction analyses were not performed in this study, highlighting the importance of considering both lipid and glycemic profiles in clinical assessments and interventions. For instance, higher ApoB levels and greater glycemic fluctuations (as indicated by SDBG and LBGI) were independently associated with an elevated risk of MDD. These observations provide a basis for future studies to evaluate whether modifying these metabolic parameters influences depressive outcomes.

These findings underscore the importance of routine screening for depressive symptoms in elderly T2D patients, particularly those with elevated ApoB levels and significant glycemic variability. Multidisciplinary approaches that integrate endocrinology and psychiatry are warranted to address the complex interplay of metabolic and mental health factors in this population.

## 5 Limitations of the study

While our study provides valuable insights, it is not without limitations. First, the retrospective design precludes causal inferences. Longitudinal studies are needed to confirm the temporal relationship between lipid dysregulation, glycemic variability, and depression. In this study, although we compared disease durations between groups to partially reduce potential reverse temporal ambiguity, causal relationships cannot be inferred from this cross-sectional design. Only patients with a longer T2D duration than MDD were included. In the future, we plan to design rigorously structured longitudinal cohort studies with extended follow-up periods to further investigate the intricate relationship between T2D and MDD. Second, our sample was limited to hospitalized elderly patients, which may limit the generalizability of our findings to community-dwelling individuals. Third, although several conventional lipid parameters were included, more detailed lipid profiling—such as LDL cholesterol subclasses, non-HDL cholesterol, or remnant cholesterol—was not comprehensively evaluated. Given the metabolic complexity of T2D, expanded lipid characterization in larger cohorts may provide additional insights into the relationship between metabolic dysregulation and comorbid MDD. Future studies incorporating broader lipid panels are warranted. Finally, although we included a broad range of lipid and glycemic indices, other factors such as inflammatory markers and neuroendocrine variables warrant further investigation. In addition, the hospital-based recruitment strategy ensured standardized assessment but may limit generalizability to ambulatory or community-based T2D populations.

## 6 Conclusion

In this study, the prevalence of comorbid MDD among hospitalized older adults with T2D was 59.31% (86/145). This proportion likely reflects the high-risk nature of a hospitalized elderly population and should not be interpreted as representative of community-based T2D cohorts. Our study also identifies ApoB, SDBG, and LBGI as factors independently associated with MDD in elderly T2D patients, emphasizing the need for a holistic approach to managing metabolic and psychological health in this population. These findings provide a theoretical basis for future research and interventions aimed at improving the long-term prognosis of elderly T2D patients at risk for depression.

## Data Availability

The raw data supporting the conclusions of this article will be made available by the authors, without undue reservation.
